# Developing and Validating Novel EHR-Based Multimorbidity-Weighted Index Across Comorbidity Measures

**DOI:** 10.1093/geroni/igaf122.3913

**Published:** 2025-12-31

**Authors:** Melissa Wei, Ashley Kang, Alexandra Klomhaus, Lucia Chen, Chi-Hong Tseng

**Affiliations:** UCLA and VA, Los Angeles, California, United States; University of California, Los Angeles, Los Angeles, California, United States; University of California, Los Angeles, Los Angeles, California, United States; University of California, Los Angeles, Los Angeles, California, United States; University of California, Los Angeles, Los Angeles, California, United States

## Abstract

Multimorbidity and physical functioning are important to measure for older adults, but tools capturing both are lacking in electronic health record (EHR) data. The multimorbidity-weighted index is weighted to physical functioning and has been validated for claims and EHR data but is not designed specifically for clinical data available in the EHR (medications, labs). Herein, we develop an EHR-based multimorbidity-weighted index (eMWI) for use in EHR systems using the Observational Medical Outcomes Partnership (OMOP) Common Data Model and validate its performance against prior comorbidity measures. To develop our 76-condition eMWI, we applied our previously validated physical functioning-based disease weights to 1) published condition algorithms when available, and 2) chart review-validated ICD-code-based definitions for remaining conditions. Using a University of California Health Data Warehouse dataset of adults ≥18 years with ≥2 outpatient encounters, 2012-2024, we compared multimorbidity values and distributions for eMWI, Charlson, Elixhauser, and simple disease count. Our final cohort included 4,672,662 adults, 55.7% female, with mean±SD age 46.3±16.8. Patients had higher multimorbidity values (mean±SD) using eMWI (5.66±7.85) vs Charlson (1.11±1.92), Elixhauser (1.89±2.51), and disease count (3±3.55). eMWI demonstrated superior performance in quantifying unique values of multimorbidity burden, exhibiting the widest range of scores (0-91) with only 26% having a score of 0. By comparison, Charlson scores ranged from 0-25 and 59% had scores of 0. We demonstrate that our newly developed eMWI can be used in EHR data for quantifying multimorbidity burden in comorbidity adjustment and risk stratification tools for adults across the lifespan, with advantages over prior measures.

